# Using three-dimensional model-based tumour volume change to predict the symptom improvement in patients with renal cell cancer

**DOI:** 10.1007/s13205-024-03967-y

**Published:** 2024-05-05

**Authors:** ChengWei Fu, JinKai Dong, JingYun Zhang, XueChao Li, ShiDong Zuo, HongTao Zhang, Shen Gao, LiJun Chen

**Affiliations:** 1 Medical School of Chinese PLA, No. 28 Fuxing Road, Haidian District Beijing, 100853 China; 2 Department of Urology, The Third Medical Center, Chinese PLA General Hospital, No. 69 Yongding Road, Haidian District, Beijing, 100039 China; 3 Department of Urology, The Fifth Medical Center Chinese PLA General Hospital, No. Yard 8, Fengtai East Street, Beijing, 100071 China; 4 Department of Radiology, The Fifth Medical Center, Chinese PLA General Hospital, No. Yard 8, Fengtai East Street, Beijing, 100071 China

**Keywords:** Renal cell cancer, Response Evaluation Criteria in Solid Tumours 1.1, Volumetric analysis, Axitinib, PD-1, Quality of life

## Abstract

In our recent study, we explored the efficacy of three-dimensional (3D) measurement of tumor volume in predicting the improvement of quality of life (QoL) in patients suffering from renal cell cancer (RCC), who were treated with axitinib and anti-PD-L1 antibodies. This study encompassed 18 RCC patients, including 10 men and 8 women, with an average age of 56.83 ± 9.94 years. By utilizing 3D Slicer software, we analyzed pre- and post-treatment CT scans to assess changes in tumor volume. Patients' QoL was evaluated through the FKSI–DRS questionnaire. Our findings revealed that 3D models for all patients were successfully created, and there was a moderate agreement between treatment response classifications based on RECIST 1.1 criteria and volumetric analysis (kappa = 0.556, p = 0.001). Notably, nine patients reported a clinically meaningful improvement in QoL following the treatment. Interestingly, the change in tumor volume as indicated by the 3D model showed a higher area under the curve in predicting QoL improvement compared to the change in diameter measured by CT, although this difference was not statistically significant (z = 0.593, p = 0.553). Furthermore, a multivariable analysis identified the change in tumor volume based on the 3D model as an independent predictor of QoL improvement (odds ratio = 1.073, 95% CI 1.002–1.149, p = 0.045).In conclusion, our study suggests that the change in tumor volume measured by a 3D model may be a more effective predictor of symptom improvement in RCC patients than traditional CT-based diameter measurements. This offers a novel approach for assessing treatment response and patient well-being, presenting a significant advancement in the field of RCC treatment.

## Introduction

Renal cell cancer (RCC) is the most common primary tumour located in the kidney, accounting for up to 85% of all cases (Fleckenstein et al. 2016). Although the 5-year survival rate of RCC has improved in recent years, the overall prognosis is still poor, especially for patients with late-stage disease (Baidya et al. 2020). Currently, various combinations of highly selective tyrosine kinase inhibitors (TKIs), such as axitinib, and immune checkpoint inhibitors (ICIs), including avelumab, nivolumab and pembrolizumab, have been approved to treat adult patients with advanced RCC (Leslie et al. 2020). The most widely used criteria for evaluating solid tumour treatment response is the Response Evaluation Criteria in Solid Tumours (RECIST) guidelines (version 1.1) (Doemel et al. 2021). However, this presents a one-dimensional (1D) measurement system that has various problems, including the difficulty in determining the diameter of irregular masses and discrepancies in the scan planes (Tran et al. 2004; Choi et al. 2017; Doemel et al. 2021). Previous studies have revealed that three-dimensional (3D) measurement has several advantages over 1D measurement, including a more accurate assessment of tumour changes and superior measurement of an irregular mass (Choi et al. 2017). For example, Doemel et al. (2021) found that the 3D assessment of advanced-stage hepatocellular carcinoma had an advantage in predicting a patient’s survival chances. Elsewhere, Steger et al. (2011) revealed that the 3D assessment of lymph nodes provides an improved prognostic value of the volume compared with measuring the short axis only. Moreover, Hadjiiski et al. (2015) found that the response assessment for bladder cancer measured according to pre- and post-treatment 3D volume changes was more accurate for irregularly shaped tumours than when measured based on the RECIST criteria. The suitability of the 3D measurement of tumour volume for treatment response evaluations in patients with RCC undergoing treatment with highly selective TKIs and ICIs has not yet been reported. In this study, 3D Slicer software (version 4.8.1, Kitware Inc, NY, USA; https://www.slicer.org) was used to construct 3D models of RCC. The main aim was to assess the reliability of the 3D measurement of tumour volume for RCC response evaluations during treatment with TKIs and ICIs and to determine the association between tumour volume change and the patient’s self-reported improvement in quality of life (QoL).

## Materials and methods

### Patients

This prospective cohort study was approved by the institutional review board of our hospital. All the recruited patients signed the informed consent form and agreed to the publication of the data. The inclusion criteria were as follows: (i) patients who were pathologically diagnosed with RCC via biopsy, (ii) patients aged > 18 years, (iii) patients who had not undergone surgery or received prior systemic treatment and (iv) patients who were suitable for treatment with axitinib combined with anti-PD-1 therapy. The exclusion criteria included (i) patients who did not have a verifiable target lesion, (ii) patients who did not have baseline or follow-up computed tomography (CT) images and (iii) patients who had received additional treatment alongside axitinib and anti-PD-1 treatment.

### Treatment

The patients were administered standard-dose (5 mg) axitinib orally twice a day and an anti-PD-1 antibody (sintilimab, 200 mg) intravenously every 3 weeks or toripalimab (3 mg/kg) intravenously once every 3 weeks.

### Treatment response assessment

The CT examinations were performed at baseline (pre-treatment) and 3 months post-treatment. All of the patients underwent a four-phase helical CT scan consisting of an unenhanced phase, a corticomedullary phase (20–50-s delay), a nephrographic phase (60–70-s delay) following intravenous injection of 100–150 mL of iso-osmolar contrast material and an excretion phase (3–5-min delay). The patients’ CT scans were downloaded from the picture archiving and communication system in digital imaging and communications in medicine format. Manual volume-of-interest segmentation of all the images and a 3D reconstruction process were performed using the 3D Slicer software (Fedorov et al. 2012). The various structures of interest were segmented manually (using a contouring method) or semi-automatically (using a thresholding method), and each structure was assigned a label. The 3D reconstruction of the various anatomical structures was then implemented using the ‘merge and build’ module in 3D Slicer (triangular mesh method) (Colen et al. 2021; Zheng et al. 2021), with a focus on the renal vasculature, urinary collecting system, kidney shape and tumour features. Finally, a smoothing filter was applied to the 3D reconstruction. All image processing was performed by the same board-certified radiologist, who has more than 10 years’ experience. The patients’ treatment response was evaluated using RECIST 1.1 criteria. Here, the patients were classified into four categories according to the changes in tumour diameter. The RECIST system includes complete response (CR, total disappearance of all target lesions), partial response (PR > 30% decrease in tumour diameter), progressive disease (PD > 20% increase or 5-mm absolute increase in tumour diameter) and stable disease (SD, a response somewhere between PR and PD) (Lubner et al. 2017; Baidya et al. 2020). The patients’ treatment response based on the 3D model tumour volume was evaluated as follows: CR, total disappearance of all target lesions; PR > 65% decrease in tumour volume; PD > 73% increase in tumour volume; SD, a response somewhere between PR and PD (Fleckenstein et al. 2016; Choi et al. 2017). The relative percentage changes (RPC) in tumour diameter or tumour volume from baseline to the follow-up point were calculated as follows:$RPC\, = \,\left( {M1{-}M0} \right)/M0\, \times \,{1}00\%$ .

where *M1* is the measurement of the tumour volume or tumour diameter at 3 months following treatment and *M0* is the measurement of the tumour volume or tumour diameter at baseline.

### Quality-of-life assessment

Self-reported QoL was evaluated using the Functional Assessment of Cancer Therapy Kidney Cancer Symptom Index–Disease-Related Symptoms (FKSI–DRS) questionnaire at baseline and 3 months post-treatment. The questionnaire includes nine symptom-specific questions related to kidney tumours, which address the following issues: lack of energy, weight loss, pain, bone pain, shortness of breath, fatigue, cough, fevers and haematuria (Motzer et al. 2020). Each symptom was scored on a scale of 0–4, where 0 denotes ‘not at all’ and 4 denotes ‘very much’. A total score was obtained by summing the scores of all items, with a higher score indicating more severe symptoms. An important difference (ID) for the FKSI–DRS questionnaire was defined as the change of at least 2 points from the baseline to the follow-up point (Motzer et al. 2020). A reduction in ID from the baseline indicated a clinically meaningful improvement in QoL.

### Statistical analysis

The data were analysed using SPSS software (version 22.0) and MedCalc statistical software (version 16.2.1). The categorical data were expressed as numbers and percentages, whereas the quantitative data were expressed as mean ± standard deviation.

The kappa statistics system was used to evaluate the agreement between the 3D model analysis and RECIST guidelines for treatment response. The kappa value (κ) used to assess the agreement between the two methods was assigned as follows: poor = κ ≤ 0.20, fair = 0.21 < κ ≤ 0.40, moderate = 0.41 < κ ≤ 0.60, good = 0.61 < κ ≤ 0.80 and excellent = κ > 0.81 (Choi et al. 2017). To evaluate the sensitivity and specificity of the change in tumour diameter in relation to the change in tumour volume in predicting the meaningful QoL improvement, receiver operating characteristic (ROC) curves were created, with the corresponding area under the curve (AUC) assessed using a Delong test.

To assess the independent predictors of clinically meaningful QoL improvement, a two-step approach was used. In the first step, a univariate logistic regression model was used to evaluate the association between meaningful QoL improvement and factors such as age, gender, tumour stage, Eastern Cooperative Oncology Group (ECOG) performance status, treatment protocol, change in tumour diameter and change in tumour volume. In the second step, all the variables that correlated with meaningful QoL improvement (*p* < 0.05) were included in a multivariate logistic regression model. A *p*-value of < 0.05 was considered statistically significant.

## Results

A total of 31 patients with RCC were screened between January 2020 and March 2022. Among them, 13 were excluded due to insufficient imaging (*n* = 4) or the absence of a verifiable target lesion (*n* = 5) or because they were receiving additional treatment alongside the axitinib/anti-PD-1 treatment (*n* = 4). This left a total of 18 patients with RCC (10 men and 8 women; mean age = 56.83 ± 9.94 years), who received the combined axitinib–anti-PD-L1 treatment for at least 3 months. At baseline, 11 (61.11%) patients were assigned an ECOG performance score of 0, whereas 7 (38.89%) were assigned a score of 1. All the recruited patients were histologically confirmed to have clear-cell RCC. The characteristics of the 18 patients are shown in Table 1.

**Table 1:** Patients’ baseline characteristics (n = 18)

Characteristics	Data
Gender [n(%)]	
Male	10 (55.56)
Female	8 (44.47)
Age (years, mean ± SD)	56.83 ± 9.94
ECOG performance status [n(%)]	
0	11 (61.11)
1	7 (38.89)
Treatment [n(%)]	
Axitinib + Sintilimab	12 (66.67)
Axitinib + Toripalimab	6 (33.33)
CT-determined T stage [n(%)]	
T3a	7 (38.89)
T3b	5 (27.78)
T3c	4 (22.22)
T4	2 (11.11)
Pre-treatment tumor diameter (cm, mean ± SD)	6.71 ± 1.57
Pre-treatment tumor volume (cm^3^, mean ± SD)	204.22 ± 99.8
Pre-treatment FKSI-DRS score (mean ± SD)	19.28 ± 6.97

*ECOG* Eastern Cooperative Oncology Group, *FKSI-DRS* Functional Assessment of Cancer Therapy-Kidney Symptom Index-Disease Related Symptoms

**Fig. 1:**
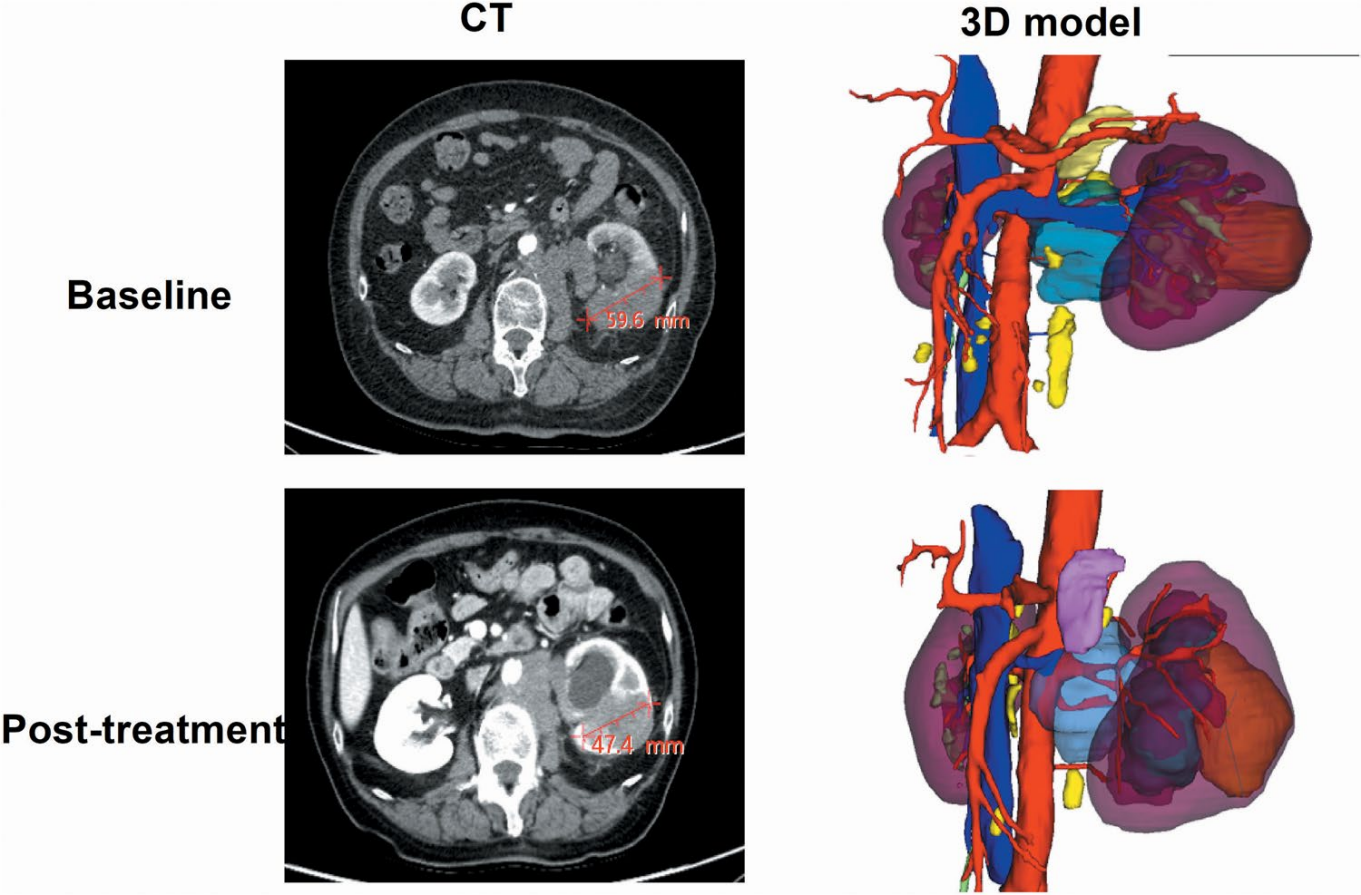
Enhanced Visualization of RCC Treatment Response in Case 1 Using CT and 3D Modeling. This figure presents a comparative view of case 1's renal cell carcinoma (RCC) before and after treatment. It includes CT images alongside a 3D virtual model. The RECIST 1.1 criteria classify the treatment response as stable disease (SD) with a slight decrease in tumor diameter. However, the volumetric criteria show an increase in tumor volume, also indicating SD. The color coding in the 3D model is as follows: artery in red, venous structures in dark blue, tumor in brown, excretory system in yellow, and normal kidney tissue in purple

**Fig. 2:**
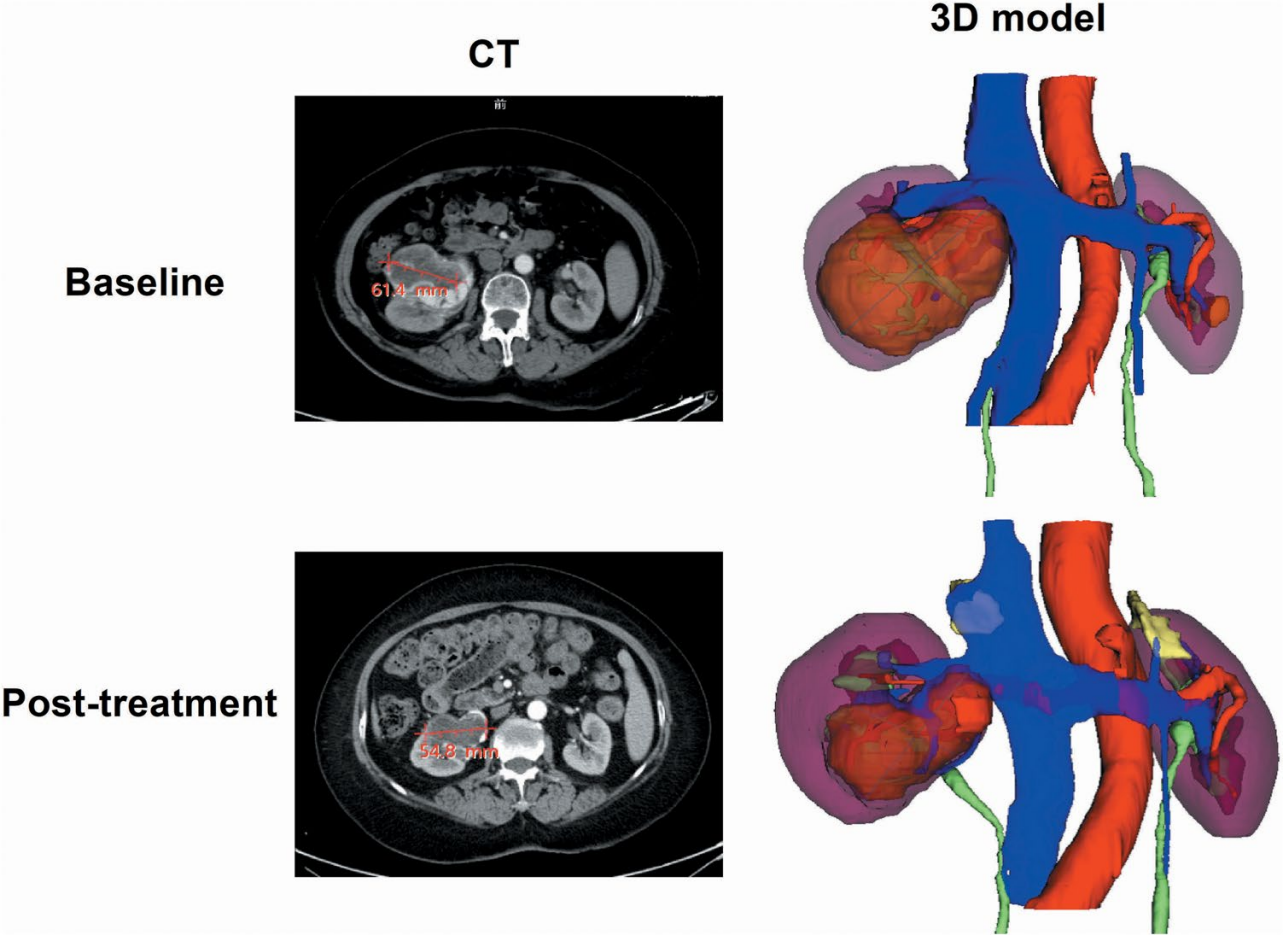
Comparative Analysis of RCC Treatment Response in Case 3 Using CT and 3D Modeling. This figure illustrates the RCC treatment response for case 3, featuring CT images and a corresponding 3D virtual model. According to the RECIST 1.1 criteria, the case is classified as SD, with a minor decrease in tumor diameter. In contrast, volumetric criteria classify the response as partial response (PR), with a significant decrease in tumor volume. The 3D model’s color scheme is as follows: artery in red, venous structures in dark blue, tumor in brown, excretory system in green, and normal kidney tissue in purple

**Fig. 3:**
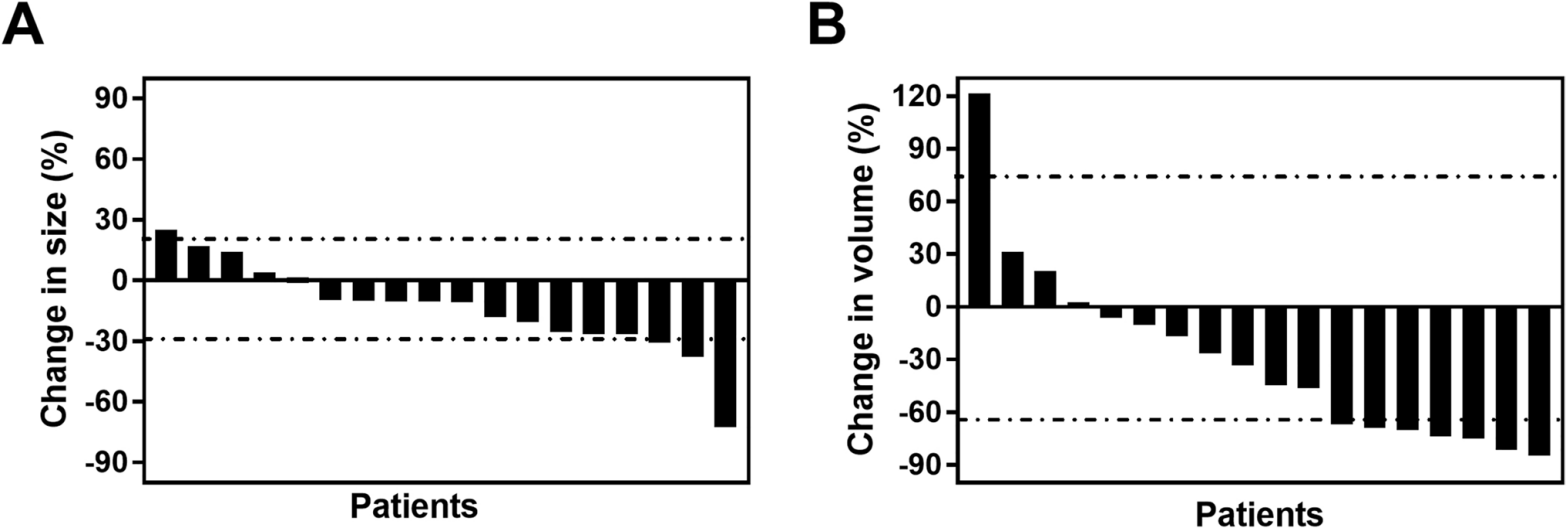
Waterfall Plot Demonstrating Changes in Tumor Diameter and Volume for 18 RCC Patients. This waterfall plot displays the changes in tumor diameter (Panel **A**) and tumor volume (Panel **B**) in 18 patients with RCC after treatment. Each bar represents an individual patient, showing the percentage change in tumor size or volume. The plot visually compares the extent of tumor shrinkage or growth post-treatment, based on RECIST 1.1 criteria for diameter and volumetric criteria for volume

The 3D models of the 18 patients were successfully constructed, and the representative images are presented in Figs. 1 and 2 (case 1 and 3). Table 2 shows the changes in tumour diameter and tumour volume indicated on the CT and 3D models. The mean diameter change in the pre- and post-treatment CT evaluation was 20.57% ± 16.19%, whereas the mean volume change in the pre- and post-treatment 3D model evaluation was 48.92% ± 32.90%. According to the RECIST 1.1 criteria, the treatment responses were CR = 0, PR = 3, SD = 14 and PD = 1 (Fig. 3A), and according to the volumetric criteria, the treatment responses were CR = 0, PR = 7, SD = 10 and PD = 1 (Fig. 3B). In 77.78% (14/18) of the patients, the same RECIST and volumetric criteria treatment response classifications were identified. After applying the volumetric criteria, the SD evaluation (RECIST) of four patients changed to a PR evaluation. There was moderate agreement between the 3D model analysis and RECIST treatment response criteria (*κ* = 0.556, *p* = 0.001).

**Table 2:** The treatment response of the 18 patients

Case No.	RECIST 1.1 criteria				Volumetric criteria			
	Baseline (cm)	Post treatment (cm)	Size change	Response	Baseline (mL)	Post treatment (mL)	Volume change	Response
1	5.96	4.74	20.47% D	SD	45.83	47.10	2.78% I	SD
2	9.43	7.03	25.45% D	SD	247.15	181.74	26.47% D	SD
3	6.14	5.48	10.75% D	SD	169.88	50.88	70.05% D	PR
4	9.80	6.10	37.76% D	PR	311.40	58.26	81.29% D	PR
5	4.45	5.20	16.85% I	SD	46.32	60.95	31.38% I	SD
6	5.08	4.56	10.24% D	SD	58.90	32.55	44.74% D	SD
7	8.24	7.45	9.59% D	SD	302.44	162.32	46.33% D	SD
8	5.68	5.12	9.86% D	SD	247.35	82.40	66.68% D	PR
9	6.21	6.45	3.86% I	SD	265.04	248.32	6.31% D	SD
10	6.83	5.04	26.65% D	SD	299.43	75.20	74.89% D	PR
11	7.45	4.32	72.45% D	PR	298.24	45.43	84.77% D	PR
12	4.56	5.70	25.00% I	PD	82.36	182.34	121.40% I	PD
13	6.78	6.08	10.32% D	SD	198.68	165.24	16.83% D	SD
14	6.23	4.32	30.66% D	PR	165.21	43.50	73.67% D	PR
15	8.14	6.67	18.06% D	SD	300.04	200.3	33.24% D	SD
16	8.45	8.34	1.30% D	SD	332.24	298.34	10.20% D	SD
17	5.26	6.02	14.45% I	SD	112.02	135.00	20.51% I	SD
18	6.13	4.50	26.59% D	SD	193.43	60.64	68.65% D	PR

*D* decrease, *I* increase, *SD* stable-disease, *PR* partial-response, *PD* progressive-disease

**Fig. 4:**
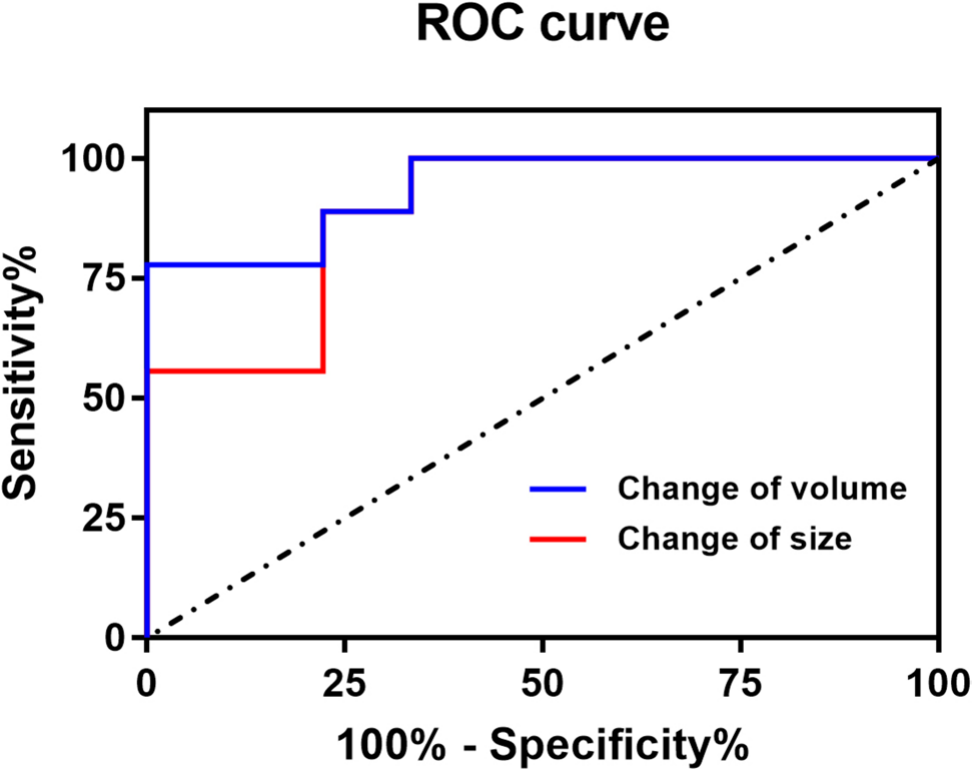
ROC Curve Analysis Comparing Tumor Diameter and Volume Changes in Predicting QoL Improvement. This figure showcases ROC (Receiver Operating Characteristic) curves evaluating the effectiveness of changes in tumor diameter versus tumor volume in predicting meaningful improvements in quality of life (QoL). The analysis compares the sensitivity and specificity of both measurement methods, with the area under the curve (AUC) indicating the predictive accuracy for each method

The mean FKSI–DRS scores at baseline and at 3 months post-treatment were 19.28 ± 6.97 and 17.67 ± 6.69, respectively. Using the FKSI–DRS scoring threshold defined in this study (ID > 2 points), nine (50%) of the 18 patients experienced a clinically meaningful QoL improvement at 3 months post-treatment. A ROC curve was used to evaluate the sensitivity and specificity of the CT-based tumour diameter change in relation to the 3D model tumour volume change to predict meaningful QoL improvement (Fig. 4). The AUCs of the CT-based tumour diameter change and the 3D-model-based tumour volume change were 0.889 [95% confidence interval (CI) 0.736–1.042] and 0.938 (95%CI 0.832–1.045), respectively. Although the 3D model-based tumour volume change had a higher AUC than the CT-based tumour diameter change, the difference in AUC between the two curves was not statistically significant (*z* = 0.593, *p* = 0.553). The multivariate logistic regression analyses indicated that the 3D model-based tumour volume change was an independent predictor of clinically meaningful QoL improvement (odds ratio = 1.073, 95%CI 1.002–1.149, *p* = 0.045).

## Discussion

In this study, using 3D Slicer software, 3D models of 18 patients with RCC were successfully constructed based on the patients’ CT images. Moderate agreement was determined between the 3D model analysis and RECIST guidelines for the treatment response (κ = 0.556). The 3D model-based tumour volume change was an independent predictor for identifying clinically meaningful QoL improvement during treatment involving TKIs and ICIs.

The 3D Slicer package is a free medical image computing software package (Fedorov et al. 2012). This software package has been used to create 3D tumour models for various solid tumours, including non-small cell lung cancer (Velazquez et al. 2013), Wilms’ tumour (Chaussy et al. 2020) and intracranial tumours (Hou et al. 2020). Three-dimensional models created using 3D Slicer have been reported to exhibit a strong correlation with the corresponding pathology (Velazquez et al. 2013). Thus, this software was used in the present study to construct 3D models of RCC. The 3D models of the 18 patients with RCC were successfully constructed based on their CT images.

Rothe et al. (2013) reported that 10%–21% of their patients received discordant RECIST and volumetric measurement response classifications, whereas in Zimmermann et al.’s study, 66%–88% of the patients received the same RECIST and volumetric measurement response classifications (Zimmermann et al. 2021). In the present study, 77.78% (14/18) of the patients received the same RECIST and volumetric criteria treatment response classifications, a result that is consistent with studies reporting other tumour types (Lubner et al. 2017; Zimmermann et al. 2021). After applying the volumetric criteria, the SD evaluation (RECIST criteria) changed to a PR evaluation in four patients. There was moderate agreement between the 3D model analysis and RECIST criteria for treatment response to TKI–ICI treatment (κ = 0.556, p = 0.001). Research has established that evaluating the treatment response on a single axial plane cannot fully reflect the attendant changes, especially in tumours with complex shapes (Doemel et al. 2021; Zimmermann et al. 2021). As such, unidimensional treatment response criteria fail to predict the survival rate of patients (Fleckenstein et al. 2016) which is likely related to the incorrect assumption that tumour diameter is directly associated with tumour volume. In this regard, 3D volume analysis has the advantage of accurately evaluating the tumour volume. Furthermore, as Xiao et al. (2015) found, tumour volume analysis is superior to the conventional technique for predicting the pathological response of rectal cancer, whereas Doemel et al. (2021) found that 3D quantitative response analysis had certain advantages over the conventional techniques in predicting the survival rate of patients with advanced-stage hepatocellular carcinoma. Fenerty et al. (2016) noted that volumetric assessment allows any tumour changes to be detected and clinical outcomes predicted earlier compared with the use of unidimensional response criteria. Overall, 3D volume analysis is potentially a more accurate predictor of the true response. Patients’ self-reported QoL could provide subjective information on the affect the disease has on individuals (Thompson et al. 2018). In terms of patients with RCC, the QoL is affected by specific disease-related symptoms, including fatigue, weakness and haematuria (Cella et al. 2018). Thus, in this study, the FKSI–DRS questionnaire was used to evaluate the patients’ QoL. Previous studies have demonstrated that the change in FKSI–DRS scores can reflect an improvement in the patient’s symptoms (Beaumont et al. 2011; Bedke et al. 2022), with Cella et al. (2018) finding that higher FKSI–DRS scores were associated with an improved overall survival rate in patients with RCC. In the present study, nine (50%) of the 18 patients experienced a clinically meaningful QoL improvement at 3 months post-treatment. Based on this and the multivariate analyses results, it was found that 3D model-based tumour volume change was an independent predictor for identifying clinically meaningful QoL improvement. Thus, 3D model-based tumour volume change may be superior to CT-based tumour diameter change in reflecting symptom improvement. However, the difference between the two ROC curves was not statistically significant, although the 3D model-based tumour volume change did have a higher AUC. The small sample used in this study may have contributed to this result.

The significance of the tumour volume change as a predictor of QoL improvement lies in the fact that it reflects the true response of the tumour to the treatment, and that it is more sensitive and accurate than the tumour diameter change measured by the CT. The tumour volume change may capture the subtle changes in the tumour shape and morphology that are not detected by the tumour diameter change, which is based on the assumption that the tumour is spherical and homogeneous. Moreover, the tumour volume change may be more relevant to the patient’s symptoms and well-being, as it may indicate the degree of tumour shrinkage or growth, the pressure on the surrounding organs and tissues, and the impact on the renal function and blood circulation.

To the best of our knowledge, this study revealed, for the first time, that 3D model-based tumour volume change may be superior to CT-based tumour diameter change in reflecting symptom improvement in patients with RCC during treatment involving TKIs and ICIs. Our results support the application of 3D models in the treatment response evaluation of patients with RCC.

However, this study also has limitations. First, only 18 patients met the inclusion criteria, and the study cohort might therefore be too small to obtain a valid conclusion. The small sample size was mainly due to the strict inclusion and exclusion criteria, the short duration of the study, and the limited availability of the combined axitinib–anti-PD-L1 treatment. Therefore, more studies with larger and more diverse samples are needed to confirm the association between tumour volume change and QoL improvement in RCC patients, and to explore the potential sources of heterogeneity and bias among the existing studies. Second, the recruited patients only underwent tumour evaluation at baseline and at 3 months post-treatment. The lack of survival data made it difficult to determine which method is more reflective of the overall clinical picture. Thus, an extended follow-up time and more survival-related data are needed to confirm our findings. Furthermore, since the patients in this study were treated with a combination of axitinib and anti-PD-1 agents, the treatment response for these patients had to be evaluated using the RECIST criteria. However, if a patient is classified as having ‘unconfirmed progressive disease’ based on these criteria, the result should be re-assessed in a dedicated earlier follow-up after 4–8 weeks (Persigehl et al. 2020). The follow-up time of this study did not meet this requirement.

## Conclusions

In this study, the 3D models of 18 patients with RCC were successfully constructed based on their CT images using 3D Slicer software. Moderate agreement was determined between the 3D model analysis and RECIST criteria for treatment response evaluation. The 3D model-based tumour volume change is an independent predictor for identifying clinically meaningful QoL improvement during TKI–ICI treatment. Thus, 3D model-based tumour volume change is potentially superior to CT-based tumour diameter change in reflecting symptom improvement in patients with RCC.
